# Deliberative Improvement of Oral Care Quality: The Horizon Europe DELIVER Project

**DOI:** 10.1177/23800844231189484

**Published:** 2023-08-11

**Authors:** S. Listl, N. Bostanci, M. Byrne, J. Eigendorf, G. van der Heijden, M. Lorenz, P. Melo, K. Rosing, P. Vassallo, E. B. van Veen

**Affiliations:** 1 Department of Dentistry–Quality and Safety of Oral Healthcare, Radboud University Medical Center–Radboud Institute for Health Sciences (RIHS), Nijmegen, Gelderland, the Netherlands; 2 Medical Faculty, Section for Translational Health Economics, Department of Conservative Dentistry, Heidelberg University, Heidelberg, Baden-Württemberg, Germany; 3 Department of Dental Medicine, Section of Oral Health and Periodontology, Division of Oral Diseases, Karolinska Institutet, Huddinge, Stockholm, Sweden; 4 Division of Dentistry, School of Medical Sciences, Faculty of Biology, Medicine and Health, University of Manchester, Manchester, UK; 5 aQua Institut, Goettingen, Niedersachsen, Germany; 6 Oral Public Health Department, Academic Centre for Dentistry Amsterdam, University of Amsterdam and Vrije Universiteit, Amsterdam, Netherlands; 7 Instituto de Saúde Pública da Universidade do Porto, Porto, Portugal; 8 Department of Odontology, Section for Oral Health, Society and Technology, Research Area Community Dentistry, Faculty of Health and Medical Sciences, University of Copenhagen, Copenhagen, Denmark; 9 Ministry for Health, Health Promotion and Disease Prevention Directorate, Valetta, Malta; 10 MLC Foundation, Utrecht, Den Haag, the Netherlands

**Keywords:** quality improvement, oral health, social determinants of health, health policy, stakeholder participation, citizen science

## Abstract

DELIVER (DELiberative ImproVEment of oRal care quality) is a multinational project funded under the EU’s Horizon Europe program; we aim to develop a blueprint model for improving the quality of oral health care for everyone: the DELIVER Quality Improvement Model. We are a team of partners from Denmark, England, Germany, Malta, the Netherlands, Portugal, and Sweden who will use a mixed-methods research approach to create a synergistic problem-solving ecosystem to convert deliberative dialogues into meaningful support for improving the quality of oral health care. Together with citizens/patients, providers, payers, and policymakers, new quality improvement (QI) approaches will be codeveloped and coproduced in 3 phases (over 4 y): phase 1 involves defining quality of oral health care, situational analysis of the status quo of QI, and consenting of core quality indicators for a Europe-wide monitoring framework. Phase 2 involves in-depth analysis of select QI approaches: 1) QI in dental practices based on patient-reported outcome and experience measures (PROMs/PREMs), 2) community-based QI for vulnerable groups, and 3) quality-oriented commissioning of oral health services. In addition, the regulatory determinants of oral health care QI will be scrutinized. In phase 3, the knowledge gained from phases 1 and 2 will be used to develop the DELIVER Quality Toolkit, a resource containing manuals and digital tools to support context-specific implementation of oral health care QI. DELIVER is anticipated to contribute to positive changes in oral health systems.

**Knowledge Transfer Statement:**

The EU DELIVER project aims to enhance the quality of oral health care through codevelopment and coproduction of solutions together with citizens/patients, providers, and policymakers. The unique multicountry nature of the project will facilitate fast-track prototype development and testing of innovative QI approaches in select countries. Reflective learning regarding the transferability of findings between different countries and settings offers unique opportunities to drive progress toward context-specific implementation of innovative oral health care QI approaches. The collective knowledge gained from the 7 European countries involved in DELIVER can also generate knowhow for improving the quality of oral health care in other countries around the globe.

## Why DELIVER?

Despite the vast disease and economic burden to individuals and society (World Health Organization 2022), a low sense of urgency for oral health care quality improvement (QI) persists in policy and practice. According to a recent working definition, quality of oral health care comprises the following 7 domains: patient safety, effectiveness, efficiency, patient-centeredness, equitability, timeliness, and access to care (Righolt et al. 2020).

The threats in oral health care quality are vast and span multiple stakeholders, multiple sectors, and multiple levels of the oral health system (practice, community, and policy levels). Oral health care is the most frequent type of health care that Europeans forgo due to financial reasons, with large inequalities in unmet dental care needs between low- and high-income groups and out-of-pocket expenditures making up 59% of total dental care expenditure (Winkelmann et al. 2022). For those who cannot access or afford oral health care, this can lead to impairment of speaking and chewing ability, psychosocial well-being, and even life-threatening systemic infections. For those who can access and afford oral health care, quality concerns include adverse effects due to diagnostic and treatment errors or medication lapses such as antibiotic overprescribing.

A complex systems problem exists in which research–policy gaps and research–practice gaps trigger inertia and inaction instead of addressing the largely preventable burden of oral diseases (Listl et al. 2022). Solving such complex issues requires coherent quality improvement (QI) strategies and efforts that span across multiple stakeholders, sectors, and levels (Kruk et al. 2018). So far, however, there is a lack of synergistic problem solving to improve the quality of oral health care together with citizens/patients, providers (oral health professionals and professionals from other disciplines), and policymakers (Listl et al. 2022).

## DELIVER’s Aims

**Figure 1.**
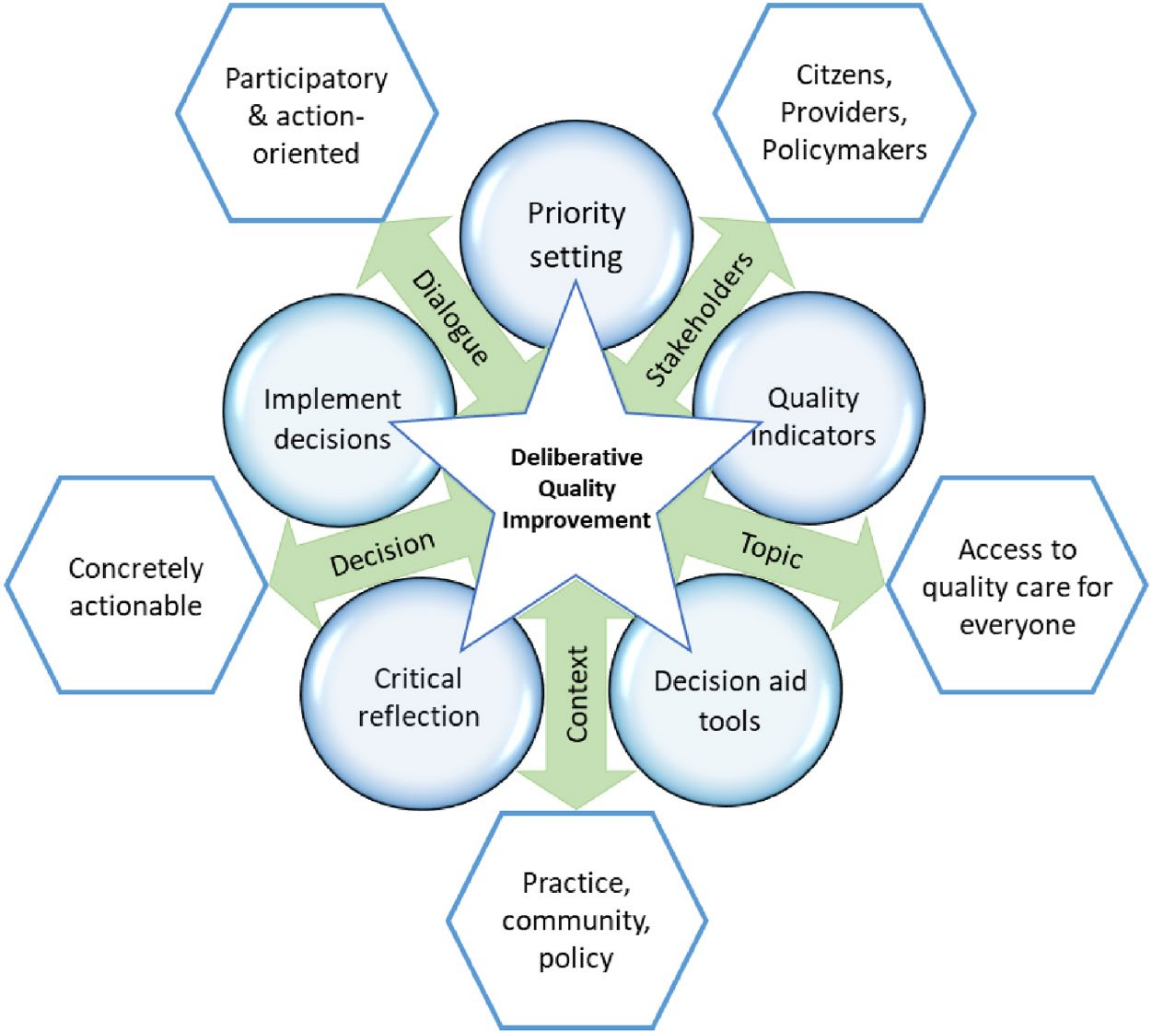
DELIVER Quality Improvement Model.

The overarching aim of DELIVER is to convert deliberative dialogues into meaningful QI for oral health care. The ultimate deliverable will be the DELIVER Quality Toolkit, which serves to operationalize the conceptual DELIVER Quality Improvement Model (see Fig.1). The DELIVER Quality Toolkit will contain actionable knowledge and implementation support tools for oral health care QI.

**Figure 2.**
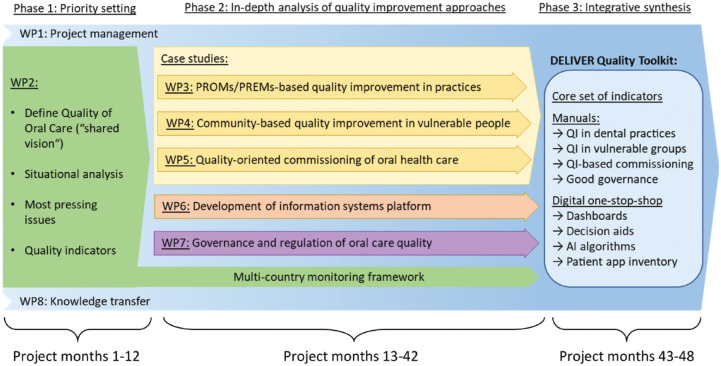
Phases and Work Packages (WPs) of the DELIVER project.

The specific objectives of the DELIVER project and their corresponding Work Packages (WPs) are as follows:

- **Objective 1—Priority setting** (corresponding to project phase 1 as described below; also see Fig. 2): the meaning of the term *quality of oral health care* will be defined. Situational analyses will explore the social worlds and arenas of action for QI in Europe. A core set of quality indicators will be consented for applicability on the practice, community, and policy levels and will subsequently feed into a Europe-wide monitoring framework (WP2).
- **Objective 2—In-depth analysis of select QI approaches** (project phase 2): building on the status quo of QI in oral health care (see objective 1 above), DELIVER will carry out multicountry case studies to develop and test novel QI interventions, comprising 1) QI in dental practices based on patient-reported outcome/experience measures (PROMs/PREMs) (WP3), 2) community-based QI in vulnerable groups (WP4), and 3) quality-oriented commissioning of oral health services (WP5). An information systems platform (WP6), insights into the governance and regulation of oral health care QI (WP7), and a multicountry monitoring/evaluation framework (WP2) will support all QI approaches throughout the project.
- **Objective 3—Integrative synthesis of knowledge** (project phase 3): the knowledge gained throughout the project will be used to develop the DELIVER Quality Toolkit, comprising manuals and digital tools for concretely actionable and context-adaptive approaches for oral health care QI (WP8).

To address these objectives, DELIVER will build on previous learnings from the development and testing of QI interventions in other fields of medicine and, where appropriate, also develop novel QI interventions. Further details relating to the objectives described above are provided in the following section below.

## DELIVER’s Methods

The complexity of aligning processes across multiple stakeholders, multiple health-related sectors (including public health and personal care delivery, insurance sector, social work sector, etc.), and multiple levels of the oral health system (practice, community, and policy levels) necessitates a shared vision and codevelopment of concretely actionable solutions for oral health care QI. To this end, DELIVER builds upon and synthesizes across state-of-the-art QI and implementation science methods. To strengthen capacities for oral health care QI, DELIVER will engage citizens/patients, providers, and policymakers in the codesign, codevelopment, and coevaluation of interventions. More specifically, DELIVER leverages the following methods throughout 3 phases over the 4 y with various WPs (also see Fig. 2):

**Phase 1: Priority setting (also see Fig. 2)**

- **Defining oral health care quality (WP2)**: the World Café methodology will be leveraged to codevelop a definition of oral health care quality together with citizens/patients, providers, and policymakers.
- **Situational analysis (WP2)**: DELIVER will decipher the social worlds and arenas of action for oral health care QI with focus on 1) practice level, 2) community level, 3) national policy level in DELIVER partner countries, and 4) wider European and global policy levels. Semistructured interviews will be held among key stakeholders (citizens/patients, providers, policymakers, experts) whose involvement will be facilitated via DELIVER’s network of partners/collaborators. The findings will help understand the context for oral health care QI and inform subsequent DELIVER steps.
- **Prioritization of pressing issues (WP2):** The nominal group technique will be used to facilitate structured group brainstorming toward prioritization of the most pressing issues for oral health care quality in Europe.
- **Consenting of quality indicators (WP2):** a modified RAND/UCLA appropriateness method will be applied to consent a multicountry harmonized set of oral health care quality indicators. This will build on previously developed sets of quality measures and include subsets that are tailored for QI activities on the practice, community, and policy levels. Multiple key stakeholders will be involved (citizens/patients, providers, policymakers, academic experts).

**Phase 2: In-depth analysis of select QI approaches (also see Fig. 2)**

- **PROM/PREM-based QI in dental practices (WP3):** adapting previously developed QI approaches from other areas of medicine, an oral health–specific PROMs/PREMs-based performance feedback intervention (based on existing questionnaires) will be developed and tested. A key component of the intervention will be a practice-level dashboard that will be tailored to visualize variations in quality indicators across different dental practices on the basis of PROMs/PREMs. This dashboard will also offer practitioners actionable recommendations based on the PROMs/PREMs provided by their patients. The specification of indicators will follow the DELIVER set of practice-level quality indicators (see above). In further case studies, the use of natural language processing to enhance information from provider rating portals will be assessed. Finally, a realist review will assess the role of patient empowerment apps to better integrate medical and oral health care.
- **Community-based QI in vulnerable population groups (WP4):** DELIVER will develop and test a prototypic community-based QI intervention. A group of citizens who benefit less from existing oral health systems and diverse community members (e.g., social workers, local public health authorities) will be convened to codesign structures and processes for deliberative problem-solving in multisectorial QI groups.
- **Quality-oriented commissioning of oral health care (WP5):** while limited resources are frequently named bottlenecks by actors working on the practice and community levels, these actors are rarely involved in resource allocation considerations. To this end, DELIVER’s resource allocation case studies will develop and test, together with citizens/patients, providers, and policymakers, a stepwise approach to oral health care resource allocation.
- **Development of information systems platform (WP6):** an integrated information system will be developed and implemented to support data collection, processing, and visualization according to the specific requirements in related work packages (WP2, WP3, WP4, WP5, WP8). This includes dashboards and decision aid tools for QI on the practice, community, and policy levels.
- **Governance and regulation of oral health care quality (WP7):** as regulations can have desirable or undesirable effects on oral health care quality, DELIVER will assess the regulatory aspects concerning oral health care quality and seek to leverage this knowledge for QI. In addition, as payment systems are a particularly relevant lever for QI, DELIVER will conduct a realist synthesis to identify the configurative elements of provider payment reforms for oral health care in European countries.
- **Multicountry monitoring/evaluation framework (WP2):** given the absence of a Europe-wide harmonized framework for monitoring and evaluation of oral health care quality, DELIVER will systematically map and assess the appropriateness of existing sources of population-level data and registry/claims data for cross-country comparability of oral health care quality. In addition, an online survey of citizens’ perception of oral health care quality will be carried out.

Different study populations will be involved in various parts of the project. In particular, to extract maximum value of information during the project period, the case studies to test QI interventions (WP3–WP5) will take place in the Netherlands, England, Denmark, and Germany while all project participants will critically assess the cross-country transferability of respective findings. This serves to ensure practical feasibility and efficient knowledge generation within the 4-y project period.

**Phase 3: Integrative synthesis of knowledge (also see Fig. 2)**

- **Knowledge transfer (WP8):** to maximize collective knowledge production and societal impact, the DELIVER consortium will actively engage in dissemination, exploitation, and communication activities throughout the entire 4-y project period. Toward the end of the project, the DELIVER consortium will convene for a participatory workshop to reflect on findings from the various workstreams and agree on how best to consolidate the collective learnings into its final product: the DELIVER Quality Toolkit (also see next section). In multidisciplinary focus groups involving researchers, educators, citizens/patients, oral health professionals/providers, and policymakers, the preferred content and packaging of end-user manuals and digital tools will be agreed. As a key outcome of the project, teaching goals for QI in dental curricula will be consented together with dental educators. Particular attention will be paid to the cross-country adaptability of the various toolkit components. To boost the future take-up and impact, the DELIVER Toolkit materials will be integrated in and officially launched via the DELIVER project website.

Note that DELIVER employs a mixed-methods approach to leverage and combine both qualitative and quantitative information for enriched insights into oral health care QI. For example, the consenting of quality indicators will draw from qualitative insights. These quality indicators will then inform the quantitative measurement of feedback information within case studies to test QI interventions (e.g., PROMs/PREMs for performance feedback interventions in dental practices) while the subsequent evaluation of the QI interventions itself will draw from case study participants’ qualitative insights. For further references to support the use of the described methodologies (particularly in relation to stakeholder engagement and patient feedback information), the interested reader is referred to previous literature (Listl et al. 2022).

## DELIVER’s Expected Results and Impacts

DELIVER’s key results are expected to be provided via the DELIVER Quality Improvement Toolkit (WP8), which integrates the key learnings and knowledge products from WP2 to WP7:

- Core set of consented quality indicators on practice, community, and policy levels (WP2)
- Knowhow for PROMs/PREMs-based QI in dental practices (WP3)
- Knowhow for intersectoral oral health care QI in vulnerable communities (WP4)
- Knowhow for quality-oriented oral health care resource allocation (WP5)
- Information system (1-stop shop): dashboards, decision aid tools (from WP6)
- Knowhow for governance/regulation of oral health care quality (WP7)

In the longer run, the implementation of DELIVER’s results is expected to have substantial scientific, economic, and societal impacts. In particular, the outputs of DELIVER are expected to provide tools to help improve the governance, financing, and delivery arrangements of oral health systems, including a shift to people-centered oral health care, better QI training of oral health care providers, and improved access and affordability of oral health care for vulnerable population groups. Ultimately, DELIVER is expected to contribute to strengthening oral health systems and to help achieve universal health coverage (World Health Organization 2022).

## It’s Time to DELIVER

In conclusion, the EU-funded DELIVER project addresses highly relevant and urgent knowledge and implementation gaps to improve the quality of oral health care. The unique multicountry nature of the project will facilitate fast-track prototype development and testing of innovative QI approaches in select countries and allow for rapid adaption for adoption in different contexts. The reflective learning regarding the transferability of findings between different countries and settings, achievable only with such international collaboration, is expected to drive progress toward context-specific implementation of innovative oral health care QI approaches in the EU and worldwide.

## Author Contributions

S. Listl, contributed to conception, design, drafted and critically the revised manuscript; N. Bostanci, M. Byrne, J. Eigendorf, G. van der Heijden, M. Lorenz, P. Melo, K. Rosing, P. Vassallo, E. B. van Veen, contributed to conception, design, critically revised the manuscript. All authors gave final approval and agree to be accountable for all aspects of the work.
